# Functional Characterization of ECP-Heparin Interaction: A Novel Molecular Model

**DOI:** 10.1371/journal.pone.0082585

**Published:** 2013-12-11

**Authors:** Ta-Jen Hung, Noboru Tomiya, Tse-Hao Chang, Wen-Chi Cheng, Ping-Hsueh Kuo, Sim-Kun Ng, Pei-Chun Lien, Yuan-Chuan Lee, Margaret Dah-Tsyr Chang

**Affiliations:** 1 Institute of Molecular and Cellular Biology, National Tsing Hua University, Hsinchu, Taiwan, Republic of China; 2 Department of Biology, Johns Hopkins University, Baltimore, Maryland, United States of America; 3 Department of Medical Science, National Tsing Hua University, Hsinchu, Taiwan, Republic of China; NCI-Frederick, United States of America

## Abstract

Human eosinophil cationic protein (ECP) and eosinophil derived neurotoxin (EDN) are two ribonuclease A (RNaseA) family members secreted by activated eosinophils. They share conserved catalytic triad and similar three dimensional structures. ECP and EDN are heparin binding proteins with diverse biological functions. We predicted a novel molecular model for ECP binding of heparin hexasaccharide (Hep6), [GlcNS(6S)-IdoA(2S)]_3_, and residues Gln^40^, His^64^ and Arg^105^ were indicated as major contributions for the interaction. Interestingly, Gln^40^ and His^64^ on ECP formed a clamp-like structure to stabilize Hep6 in our model, which was not observed in the corresponding residues on EDN. To validate our prediction, mutant ECPs including ECP Q40A, H64A, R105A, and double mutant ECP Q40A/H64A were generated, and their binding affinity for heparins were measured by isothermal titration calorimetry (ITC). Weaker binding of ECP Q40A/H64A of all heparin variants suggested that Gln^40^-His^64^ clamp contributed to ECP-heparin interaction significantly. Our *in silico* and *in vitro* data together demonstrate that ECP uses not only major heparin binding region but also use other surrounding residues to interact with heparin. Such correlation in sequence, structure, and function is a unique feature of only higher primate ECP, but not EDN.

## Introduction

Eosinophil granulocyte, a multifunctional leukocyte generated from bone marrow, involves in allergic, parasitic and chronic inflammatory diseases, and serves as a key mediator in allergy and asthma [1,2]. During inflammation, eosinophil granulocyte secretes four primary granular proteins including eosinophil cationic protein (ECP), eosinophil derived neurotoxin (EDN), major basic protein and eosinophil peroxidase [3]. ECP and EDN were first isolated from patients with marked peripheral blood eosinophilia using heparin-Sepharose column chromatography in 1986 [4]. Both shares specific sequence homology and tertiary structure with human pancreatic ribonuclease (RNase1), thus are classified into human RNaseA superfamily and respectively named as human RNase2 (EDN) and RNase3 (ECP) [4]. Mature ECP and EDN are 15-16 kDa polypeptides composed of 133 and 134 amino acids [5,6]. Similar to all human RNase family members except RNase5, ECP and EDN have 8 cysteines forming 4 pairs of disulfide bonds in three dimensional structures [7]. In addition, they have conserved catalytic triads including a Lys fitting CKXXNTF (where X represents any amino acid) motif and two His within conserved sequences FXXQH and PVHXD [7]. 

Among all human RNaseA family members, ECP and EDN share the most sequence conservation with 67% identity and 76% similarity. However, their selective biological activities are quite different, for example, EDN has comparable ribonucleolytic activity as human RNase1 [8], whereas ECP exhibits only 1% ribonucleolytic activity of EDN [9]. ECP is extremely toxic to a wide range of pathogens including helminthes [10], bacteria [11] and virus [12]. Besides, it also inhibits the growth of mammalian cells [13]. On the other hand, EDN has neurotoxicity [14] and antiviral activity [15] but is relatively ineffective against helminthes [10]. Differential functions of ECP and EDN have been attributed to their unique sequence and structural features [16].

ECP and EDN are cationic proteins respectively containing 20 (19 Arg and 1 Lys) and 12 (8 Arg and 4 Lys) basic amino acids leading to high isoelectricpoints of 10.8 and 8.9, which promotes electrostatic interactions with negatively charged molecules. ECP interacts with cell surface glycosaminoglycans (GAGs), especially heparan sulfate proteoglycans, which mediates lipid raft-dependent macropinocytosis [17]. Since heparin/heparan sulfate (HS) are the main GAGs integrated in extracellular matrix [18], GAG recognition might be the first step for cytotoxic effect of ECP, and subsequently asthma [19] and other inflammatory diseases [20]. Recently sequence motif ^34^RWRCK^38^ located at surface loop3 of ECP was identified as a major heparin binding region (HBR) [21]. Two more segments ^73^RSRFR^77^ and ^101^RPGRR^105^ were also predicted to be HBRs. Besides, *in silico* comparison of these three regions on ECP to corresponding regions in RNase1-13 demonstrated that the ^34^RWRCK^38^ was a unique peptide motif [22]. Notably, mutant recombinant ECP with Ala replacing all basic residues in 3 HBRs still possessed 19% binding activity to low molecular weight heparin (LMWH), suggesting that additional residues or factors are operative in the ECP-LMWH interaction [22]. 

Electrostatic interactions between negatively charged sulfo/carboxyl groups on heparin/HS and positively charged residues on heparin binding proteins such as antithrombin III and interleukin-8 are well known [23]. Nevertheless, hydrogen bonding, van der Waals (vDW) forces and hydrophobic interactions can be also involved in the interaction. Non-ionic interactions, for example, contribute to the majority of free energy for the interaction between basic fibroblast growth factor and LMWH, and ionic interactions contribute only 30% of the free energy [24]. Moreover, polar residues such as Asn and Gln in heparin binding regions are often observed to form hydrogen bonds with backbone of polysaccharide [25]. The structure of recombinant ECP has been solved by X-ray crystallography [26,27] and NMR [28]. Besides, complex structures of ECP with sulfate anions [29] and 2’, 5’-ADP [30] are also resolved. In 2010, major heparan sulfate binding sites on refold ECP were resolved by NMR, pointing out that a central cavity including helix α1 (Ala^8^–Gln^14^), loop3 (Tyr^33^–Arg^36^), strand *β*1 (Gln^40^–Leu^44^), and strand *β*6 (His^128^–Asp^130^) is crucial to accommodate ECP [31]. Recently, ECP structure in complex with trisaccharide heparin mimetic resolved by NMR indicated the contribution of helix α1 (Arg^7^, Gln^14^, and His^15^), loop3 (Arg^34^−Asn^39^), strand *β*1 (Gln^40^), loop4 (His^64^), and strand *β*6 (His^128^) [32]. These results strongly suggest that ECP contributes not only by positive charged residues but also by Asn and Gln to stabilize heparin derivatives in a cavity surrounded by N-terminus, loop3, loop6 and loop7.

In this study contributions of amino acids in ECP-heparin hexasaccharide (Hep6) interaction were estimated using molecular docking simulation. *In silico* analysis with flexible side-chain of basic and polar residues suggested that a clamp-like structure formed by Gln^40^ and His^64^ was present only in ECP- Hep6 interaction but not in corresponding residues of EDN. Besides, the involvements of Gln^40^ and His^64^ in ECP-heparin interaction were verified by *in vitro* thermodynamic analysis. Requirement of sulfo groups on heparin for binding to ECP was also demonstrated. Finally, docking simulation of ECP with heparin dodecasaccharide (Hep12) suggested that other residues outside the basic cavity were also participated in long-chain heparin binding.

## Materials and Methods

### Materials

Monoclonal mouse anti-ECP (EG2) antibody was purchased from Diagnostics Development A P&M Venge AB-company (Sweden) and goat anti-mouse horseradish peroxidase antibody was purchased from Jackson ImmunoResearch (USA). Low molecular weight heparin (LMWH), high molecular weight heparin (HMWH; heparin sodium salt) and Hep6 were purchased from Iduron (UK). *N*-acetyl heparin (NAcH), *de*-*N*-sulfated heparin (*deN*-SH), and *N*-acetyl-*de*-*O*-sulfated heparin (*N*Ac-*deO*-SH) were purchased from Sigma-Aldrich (USA). *De*-2-*O*-sulfated heparin (*de*2*O*-SH) and *de*-6-*O*-sulfated heparin (*de*6*O*-SH) were purchased from Neoparin (USA). 2-aminoacridone (AMAC) was purchased from Invitrogen (USA). Chemicals were purchased from Sigma-Aldrich (USA) unless otherwise specified.

### Fluorescence-assisted carbohydrate electrophoresis (FACE)

Heparin derivatives were individually reacted with AMAC as previously described [33]. Briefly, 50 µg each heparin derivative was incubated with 40 μl solution containing 1.25 M AMAC, 85% DMSO, and 15% acetic acid at 25 °C for 15 min. To this, 40 μl 1.25 M sodium cyanoborohydride (NaBH_3_CN) was added, and the mixture was incubated at 37 °C for 16 h. At the end of the reaction, 720 μL 95% ice-cold ethanol was added and placed at -20 °C for 15 min. The sample was centrifuged at 4 °C for 5 min at 14,000 × *g*. The supernatant was carefully discarded, and the pellet was freeze-dried by ScanVac CoolSafe (LaboGene^TM^, Denmark). The dried pellet was dissolved in sterile deionized water at an appropriate concentration for a measurement. The AMAC-labeled probes thus prepared were stored at -80 °C until use.

AMAC-labeled probes and proteins were mixed and incubated at 25 °C for 15 min. The mixture was loaded onto a 1% agarose gel plate, and electrophoresed for 20 min in a buffer containing 40 mM Tris-acetic acid, 1 mM EDTA, pH 8.0. The AMAC labeled probe was detected under UV light and the gel was scanned by QUANTUM-ST4 (Vilber Lourmat, Germany).

### Competitive cell enzyme-link immunosorbent assay (ELISA)

Confluent monolayers of Beas-2B cells (ATCC number: CRL-9609) in 96-well plate were pre-incubated with serially diluted heparin derivatives in serum-free RPMI 1640 medium at 4 °C for 30 min. Recombinant ECP was added to the cells and incubated at 4 °C for 60 min. Cells were washed with 200 μl ice-cold PBS twice, and fixed with 2% paraformaldehyde/PBS at 25 °C for 15 min. Then, 200 μl ice-cold PBS was added to wash the cells prior to blocking with 2% BSA/PBS at 25 °C for 90 min. The level of bound ECP was quantified by ELISA analysis using EG2 antibody (Diagnostics Development A P&M Venge AB-company) and a horseradish peroxidase conjugated goat anti-mouse IgG as the primary and secondary antibody, respectively. After the cells were washed by ice-cold PBS, 50 μl Super Signal^®^ West Pico Chemiluminescent Substrate (Thermo, USA) was added and chemiluminescent intensity was measured by Wallace Vector II (LS55, PerkinElmer, USA). The amount of recombinant ECP bound to cells without heparin derivatives treatment was set to 100%.

### *In silico* docking simulation of Hep6 and dodecasaccharide to ECP

Coordinates of crystal structures of human ECP and EDN were taken from: ECP, A chain of 1DYT [34]; EDN, 1GQV [35] deposited in PDB database. Citric acids and a Fe(III) ion in the complex were removed and the protein structure was energy minimized with GROMOS 96 force field. Heparin oligosaccharides with all iduronic acids in ^1^C_4_ conformation were energy minimized with MMFF94 force field. *In silico* docking experiment was conducted using AutoDock Vina program [36]. For both global and local search, side chains of the basic amino acids including Arg, Lys and His as well as Gln and Asn residues in the cavity of the molecular surface were set to be flexible. All rotatable bonds in the ligand were set to be rotatable. Global search was performed in the grid box with a dimension of 60×60×60 angstroms which was large enough to cover the entire protein surface and a bound ligand. Global search was performed at least two times with a single ligand to confirm consistency. Local search was performed in a smaller space at the predetermined position to obtain an accurate bound pose and binding energy.

### Construction, expression and purification of wild type and mutant ECPs

DNA encoding *ecp* without the signal sequence was cloned into pET23a plasmid (Novagen, USA) between the *Nde*I and *Bam*HI sites to generate pET23a-*ecp* containing a C-terminal His_6_ tag as previously described [26]. The plasmid was used as a template to generate single mutant including Q40A, H64A, and R105A as well as double mutant Q40A/H64A using QuikChange site-directed mutagenesis (Stratagene, USA). To express wild type ECP (wtECP) and its mutants, plasmid was used to transform *Escherichia coli* (*E. coli*) BL21 (DE3) CodonPlus^®^ (Novagen, USA). Two milliliters of the overnight culture was inoculated in 100 ml LB broth containing 100 μg/ml carbencillin, and incubated at 37 °C until OD_600_ reached 0.4 to 0.6, then IPTG (Ameresco, USA) was added to a final concentration of 0.5 mM before 4 h incubation at 37 °C. The bacteria were harvested by centrifugation at 2,900 × *g* for 10 min, and the pellets were homogenized by EmulsiFlex-C3 (AVESTIN, Canada). Proteins were collected from inclusion bodies that were refolded at 4 °C by dialysis in refolding buffer (20 mM Tris, 0.5 M arginine, 0.2 mM GSSG, 2 mM EDTA, 10% glycerol, pH 8.5). These purified proteins were concentrated by Amicon Ultra-15 (Millipore, USA) and stored in PBS at -80 °C until use. After purification, Endotoxin Removing Gel (Pierce, USA) was applied to remove lipopolysaccharide (LPS) before ECP storage and residual LPS level was measured by HEK-Blue™ LPS Detection Kit (InvivoGen, USA) before each treatment.

### Enzymatic activity assay of wild type and mutant ECPs

Ribonuclease activities of recombinant wtECP and mutant ECPs were measured using yeast tRNA (Invitrogen, USA) digestion method [37], using bovine pancreatic RNaseA (USB, USA) as positive control. Three hundred microliters of 100 mM sodium phosphate buffer, pH 7.4, and 500 μl diethylpyrocarbonate-ddH_2_O were mixed with 50 μl 0.05 μM RNaseA and 5 μM wtECP or each mutant ECP separately. Ten microliters of 5 mg/ml yeast tRNA was added and incubated at 37 °C for 0, 5, 10 and 15 min. Ice-cold 500 μl stop solution (1:1, v/v, 40 mM lanthanum nitrate and 6% perchloric acid) was added and shaken for 10 min to stop reaction. Intact yeast tRNA was suspended by centrifugation at 16,100 × *g* at 25 °C for 5 min. One hundred microliters of supernatant in each tube was placed on to a 96-well plate. The amount of soluble tRNA in supernatant was determined by A_260 nm_. The calculation has been described previously [12].

### Isothermal Titration Calorimetry (ITC)

Thermodynamic analysis of wtECP and mutant ECPs to bind heparin was performed using a MicroCal iTC200 calorimeter (MicroCal, Northampton, USA) [38]. ECP was dialyzed against PBS before use and the same buffer was used to prepare heparin solutions. All experiments were performed at 25 °C, using ECP (20 μM, as protein) and heparin (200 μM). The protein solution was placed in a calorimeter cell and the heparin solution was loaded into the syringe injector. In individual titration, auto-controlled microsyringe injected 2 μL heparin solution into the protein solution with an interval of 120 sec. The integrated heat change was analyzed by means of non-linear regression using the MicroCal Origin software, and enthalpy change (ΔH), dissociation constant (*K*_D_), and stoichiometry value (N = X/M) were obtained from a single sigmoidal titration curve [39]. The reported binding constants were the average of triplicate measurements. The effect of dilution of the heparin solution in the titration cell was removed by subtracting calorimetric data for a blank titration, which consisted of titration of heparin solution into PBS.

### Statistical analysis

Statistical analysis was performed using GraphPad Prism 5 (GraphPad Software, USA). All data were shown with mean ± standard deviation (SD). Statistical significance between a set of data was determined by unpaired two-tailed Student’s *t*-test.

## Results

### Sequence alignment of eosinophil RNases

**Figure 1:**
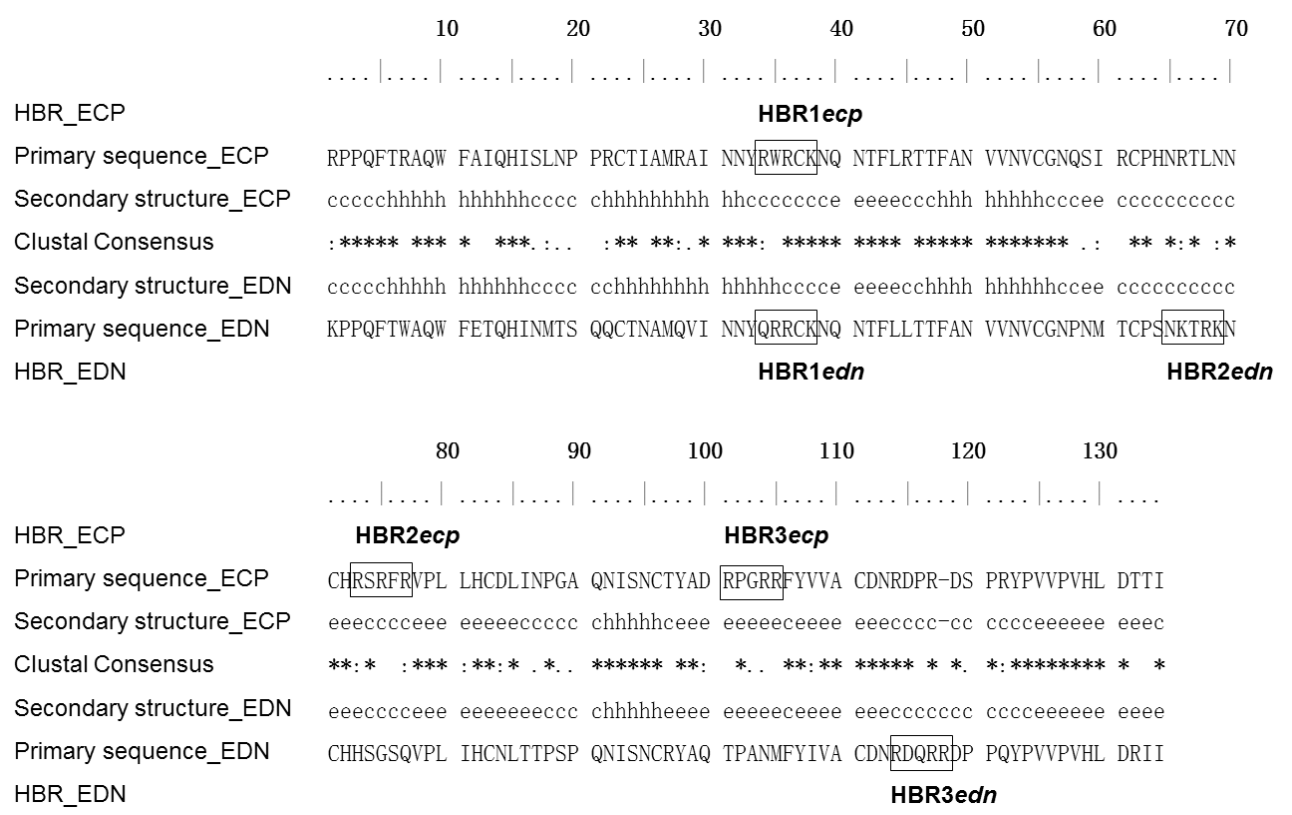
Sequence alignment of human eosinophil RNases and HBRs. Amino acid sequences of human ECP (accession number P10153.2) and EDN (accession number P12724.2) were aligned using *Clustal* *X2* [54]. Putative heparin binding regions (HBRs) were framed. Fully conserved amino acids were indicated by asterisk (*), highly similar amino acids were indicated by colon (:), and weakly similar amino acids were indicated by dot (.). Secondary structures of human eosinophil RNases were shown in the middle. Structural elements including α-helices, β-strands, and coils were represented as “h”, “e”, and “c”, respectively.

Both ECP and EDN have three putative HBRs each of which contains three basic residues within contiguous five amino acids [22]. Among these HBRs only HBR1 on ECP (HBR1*ecp*, ^34^RWRCK^38^), and HBR1 on EDN (HBR1*edn*, ^34^QRRCK^38^), were located at a comparable position in primary sequence (Figure 1). HBR1*edn* sequence fit heparin binding motif of XBBXBX, and HBR2*edn*, ^65^NKTRK^69^, also fit the same pattern, except in a reverse order [40]. However, the conventional heparin binding motif was not apparent in ECP. Secondary structure alignment (Figure 1) between ECP (PDB: 1DYT) and EDN (PDB: 1GQV) revealed that most HBRs in ECP and EDN were located in loops, except that HBR3*ecp* (^101^RPGRR^105^) was located in the β-sheet and part of HBR1*edn* (Gln^34^ and Arg^35^) was located at the α-helix structure. 

### Influence of heparin chain length in ECP binding

**Figure 2:**
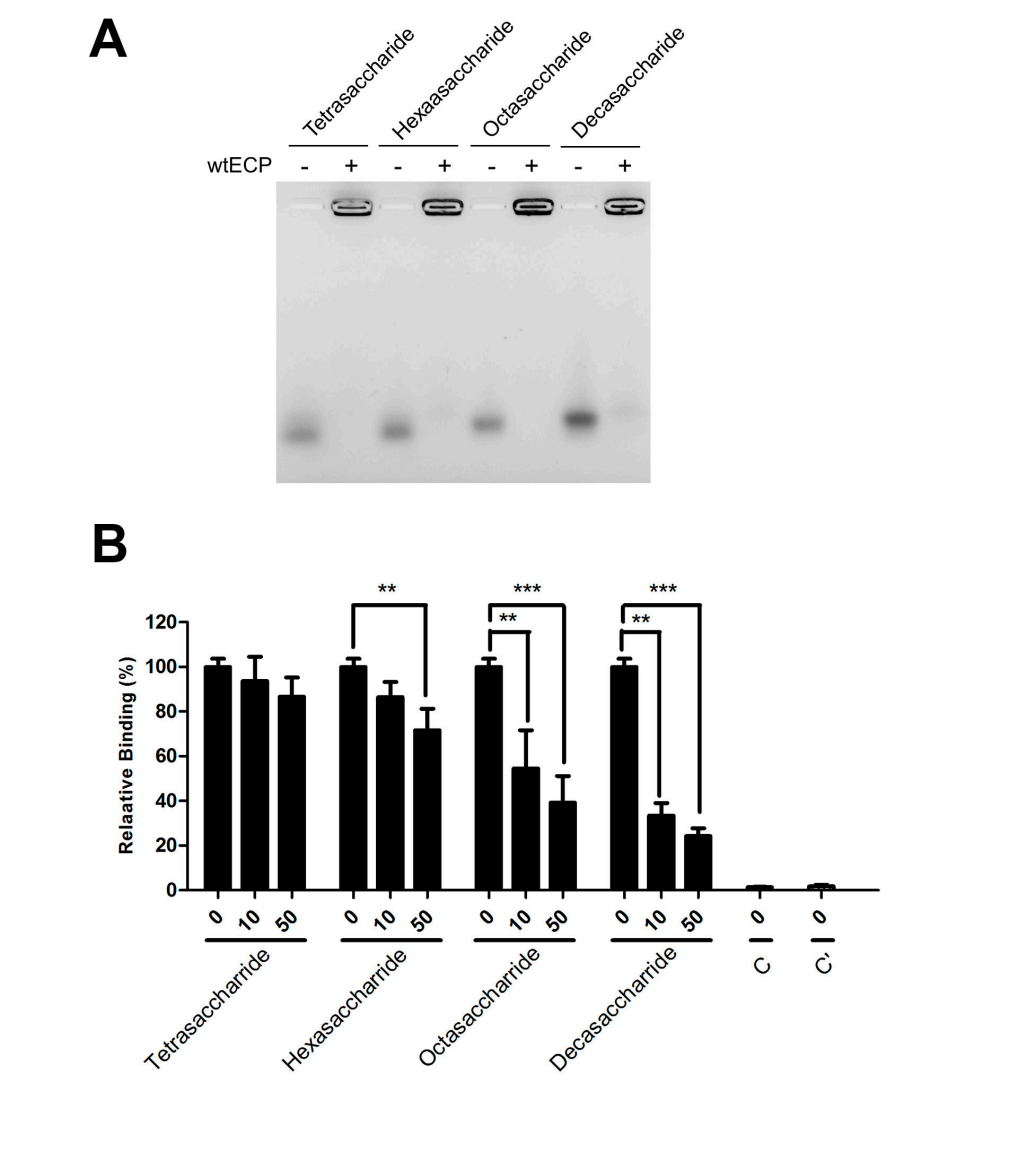
Requirement of heparin length for interaction with ECP. (A) AMAC-labeled heparin oligosaccharide (10 nmol) were incubated with or without wtECP (heparin: wtECP ratio of 5) in PBS at 25 °C for 15 min, and the reaction products were separated by gel electrophoresis using 1% agarose gel. (B) Beas-2B cells were pre-incubated with heparin oligosaccharide in RPMI 1640 medium at 4 °C for 30 min before treatment with 0.4 μM of wtECP at 4 °C for an additional 1 h. The level of bound proteins was assayed by ELISA and the amount of wtECP bound to Beas-2B cells without heparin derivative treatment was set as 100%. C, untreated. C’, treated with EG2 antibody only. The data represented three independent experiments and the error bar was shown as SD. **, *P* < 0.01; ***, *P* < 0.001.

We have previously demonstrated that a synthetic heparin oligosaccharide containing a L-idopyranosyl sugar matrix [41] and two disaccharide repeats was the minimal requirement for ECP binding [21]. However, specific interaction between ECP and natural heparin oligosaccharides remained unknown. In this study, the requirement of heparin length for interaction with ECP was investigated by FACE analysis. As shown in Figure 2A, recombinant wtECP was incubated with AMAC-labeled heparin oligosaccharides with disaccharide repeats from two to five, maintaining identical molar ratios (heparin: wtECP ratio of 5). The unbound oligosaccharides and those bound by wtECP were clearly separable, and the fluorescence intensities were analyzed. In all cases, the positions of AMAC-labeled heparin oligosaccharides shifted in the presence of recombinant ECP, suggesting that heparin tetrasaccharide satisfied the binding of heparin molecules by wtECP.

Next, the dependence of heparin length for wtECP binding to bronchial epithelial Beas-2B cells was examined by competitive cell ELISA. Figure 2B showed that at a concentration of 50 μg/ml only octasaccharide and decasaccharide showed significant inhibitory effects, respectively 61% and 76%, whereas hexasaccharide and tetrasaccharide reduced 25% and less than 20% cell binding activity of wtECP. Although the effects appeared to be concentration dependent, these results indicated that binding of heparin oligosaccharides by wtECP and Beas-2B cells are not by exactly the same mechanism. Longer chain lengths were required by the cells than ECP itself. All cell ELISA data were summarized in Table 1.

**Table 1:** Competitive cellular binding effect of heparin derivatives.

Heparin derivative	Mean ± SD (%)	
	10 μg/ml	50 μg/ml
Tetrasaccharide	93.7 ± 10.9	86.7 ± 8.6
Hexasaccharide	86.4 ± 6.9	75.4 ± 10.9
Octasaccharide	54.5 ± 17.1 (**)	39.2 ± 11.9 (***)
Decasaccharide	33.4 ± 5.6 (**)	24.2 ± 3.5 (***)
*de*-2-O-SH	48.9 ± 8.6 (***)	41.4 ± 13 (***)
*de*-6-O-SH	47 ± 8.5 (***)	35.9 ± 8.7 (***)
*de*-N-SH	52.8 ± 4.5 (***)	35.5 ± 12.6 (***)
*N*Ac-H	62.1 ± 1.3 (***)	55.5 ± 13.6 (***)
*N*Ac-*de*-*O*-SH	95.8 ± 8.2	92.4 ± 9.8
HMWH	56.8 ± 4.4 (***)	28.9 ± 1.8 (***)
LMWH	42.6 ± 9.2 (***)	33.2 ± 4 (***)

The average amount of 0.4 μM wtECP bound to Beas-2B cells was set as 100% binding. **, *P* < 0.01; ***, *P* < 0.001

### Binding of ECP to Hep6

**Figure 3:**
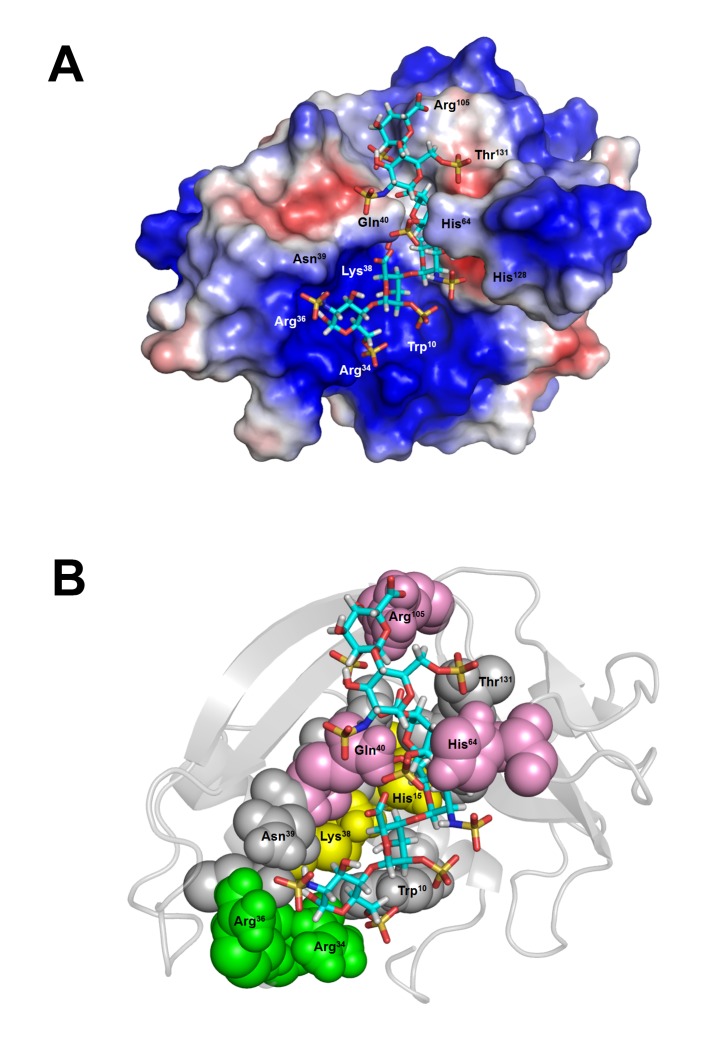
Predicted ECP-Hep6 interaction. (A) wtECP surface was colored according to the electrostatic potential. Blue, red and white color indicated respectively a region of positive (+ 67 kT/e), negative (- 67 kT/e), and neutral potential. (B) wtECP structure was presented in cartoon and the residues interacting with Hep6 were shown in colored spheres with indicated numbers: residues located at RNase active sites were in yellow, residues located at HBR1*ecp* were in green, residues with large contribution in docking simulation were in pink, and other residues were in gray. Hep6 was shown in stick and CPK color.

To investigate potential binding modes and the residues involved in the binding, docking simulation for interaction of Hep6, [GlcNS(6S)-IdoA(2S)]_3_, with wtECP and 14 mutant ECPs were performed. Our *in silico* data showed that the lowest binding energy and *K*_D_ value of Hep6 binding to wtECP was -9.24 kcal/mol, i.e., and 160 nM, respectively. In Figure 3A, the binding mode of Hep6 with the lowest energy revealed that Hep6 was located within a basic cavity on the surface of wtECP. Molecular interactions between Hep6 and wtECP including 2 ionic bonds, 6 hydrogen bonds, and mainly vDW force were listed in Table S1. In a good agreement with our previous study [21], the binding model indicated that HBR1*ecp* ^34^,RWRCK^38^, bound to Hep6 by making ionic bonds between basic residues including Arg^34^ and Arg^36^ and 6-*O*-sulfated, *N*-sulfated glucosamine residue 1 (SGN1) (Figure 3B, green spheres, Table S1). Cys^37^ also hydrogen bonded to SGN1. Regarding other putative HBRs in ECP [22], only Arg^105^ located at HBR3*ecp* ^101^,RPGRR^105^, interacted with 2-*O*-sulfated iduronic acid residue 6 (IDS6) through hydrogen bonding, whereas HBR2*ecp* ^73^,RSRFR^77^, had no interaction with Hep6 in this model. The heparin molecule also interacted with residues including His^15^ and Lys^38^ which were expected to serve as proton donors in the case of RNase activity, suggesting that Hep6 shared the same surface area with RNA for binding (Figure 3B, yellow spheres) [42]. Interestingly, the bound Hep6 was also stabilized by hydrogen bond and vDW interaction with Gln^40^ and His^64^ (Figure 3B, pink spheres, Table S1), implying that these residues might facilitate sugar binding by the predicted HBRs. 

Subsequently, each amino acid predicted to interact with wtECP (within vDW force/hydrogen bond distances) was individually changed into Ala by AutoDock, and contribution of each residue was estimated by comparing difference in Hep6 binding energy between wtECP and mutant ECP. In addition to previously reported heparin binding residues Arg^34^, Arg^36^ and Lys^38^ [21], Table S2 indicated large contributions of Gln^40^ (-0.92 kcal/mol), His^64^ (-0.54 kcal/mol) and Arg^105^ (-0.67 kcal/mol) to heparin binding, suggesting potential roles of these amino acids for stabilization of heparin oligosaccharide. 

### Comparison of putative heparin binding clamp in ECP with corresponding residues in EDN

**Figure 4:**
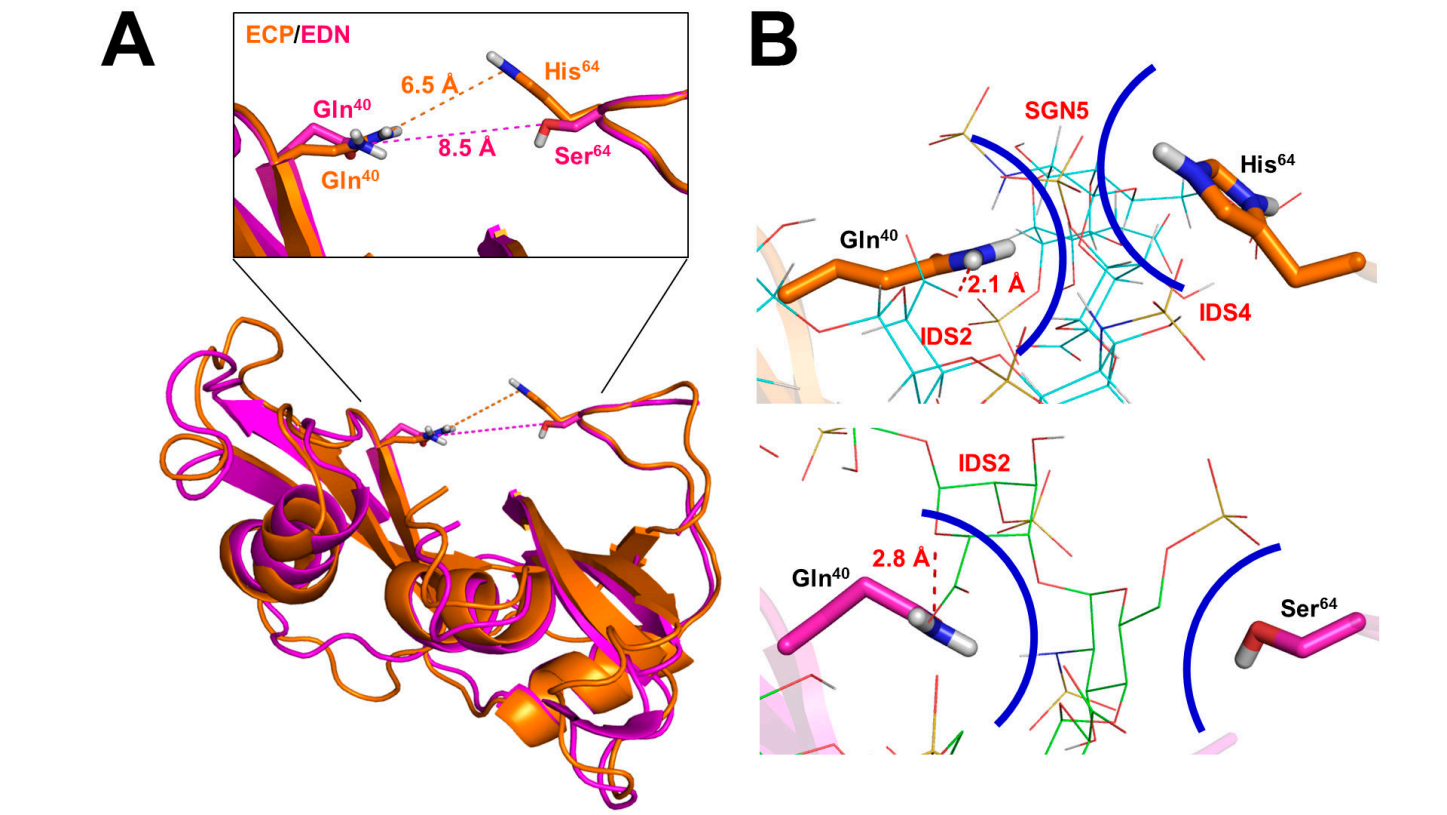
Putative heparin binding clamp in ECP with corresponding residues in EDN. (A) Superimpose analysis was performed using Lsqkab. Hep6 bound pose of ECP was superimposed on that of EDN by fitting Cα atoms of residues 40 (Gln) and 64 (His/Ser). Distances between these two residues were analyzed by PyMOL. (B) Hep6 were shown in line and CPK color. Residues were shown in orange (ECP) and pink (EDN) sticks. Hydrogen bonds were shown in red dashed lines with indicated distance. The vDW forces between the corresponding residues on ECP/EDN and the surrounding sugar moieties with indicated moiety name and number were shown as curve colored in blue indicate. SGN, 6-*O*-sulfated, *N*-sulfated glucosamine; IDS, 2-*O*-sulfated iduronic acid.

According to our previous docking simulation data (Table S3) [43], the predicted binding energy of complex EDN-Hep6 was -9.27 kcal/mol, which was in the same order as that of wtECP (Table S4). Superimposition of the Hep6 bound pose of ECP comparing to that of EDN clearly revealed resembling clefts on ECP/EDN surface, in particular a clamp comprised of residues Gln^40^ and His^64^ in ECP and comparative base pair Gln^40^ and Ser^64^ in EDN (Figure 4A). The predicted distance between Gln^40^ and Ser^64^ in EDN (8.5 Å) was 2 Å longer than that of Gln^40^ and His^64^ in ECP (6.5 Å), suggesting that the presence of such Gln^40^/His^64^ clamp in ECP only might facilitate stabilization of heparin molecule. Figure 4B showed that both Gln^40^ and His^64^ of ECP were located in vDW force contact range to interact with Hep6 and a hydrogen bond (2.1 Å) formed between the amide group of Gln^40^ and the carboxyl group of IDS2. Likewise, a hydrogen bond (2.8 Å) between the amide group of Gln^40^ and the ring oxygen of IDS2 was observed in EDN-Hep6 interaction. It should be noticed that His^64^ in wtECP formed 5 vDW interactions with SGN3, IDS4 and SGN5 (Table S1), yet Ser^64^ in EDN was too far to interact with heparin molecule (Figure 4B). In addition, contribution of Gln^40^ in EDN interaction with Hep6 (-0.77 kcal/mol) was also predicted to be higher than all other residues (Table S4), similar to the case of Gln^40^ in ECP (Table S2). 

### Expression and purification of wtECP and mutant ECPs

To investigate functional roles of Gln^40^, His^64^, and Arg^105^ in heparin binding, site-directed mutagenesis was carried out to generate 3 single mutant ECPs including Q40A, H64A, and R105A. To evaluate the importance of the binding clamp between Gln^40^ and His^64^, double mutant ECP Q40A/H64A was also generated. wtECP and mutant ECPs were expressed in *E. coli* BL21-CodonPlus^®^ (DE3) and the overexpressed proteins were purified from inclusion bodies by affinity column chromatography using Ni^2+^ Sepharose followed by refolding as previously described [44]. Figure S1 showed single band of major product of approximately 16 kDa (15% (w/v) by SDS-PAGE) in all recombinants and wtECP. RNase activities of refold wtECP and mutant ECPs were examined by yeast tRNA cleavage assay for 15 min and summarized in Table S5. The RNase activity of bovine RNaseA was 1.171 for soluble tRNA/pmol, close to the reported number of 1.208 for soluble tRNA/pmol [12]. The RNase activities of wtECP, mutant ECP Q40A, H64A, R105A and double mutant ECP Q40A/H64A were 0.0049, 0.0043, 0.0068, 0.0032 and 0.0056 for soluble tRNA/pmol, respectively. Only minor difference was seen in RNase activity between wtECP and mutant ECPs indicated that all recombinant ECPs were refolded with catalytic function, and Gln^40^, His^64^, and Arg^105^ were not crucial for enzymatic activity.

### Determination of binding affinities between wtECP/mutant ECPs and heparin derivatives

**Figure 5:**
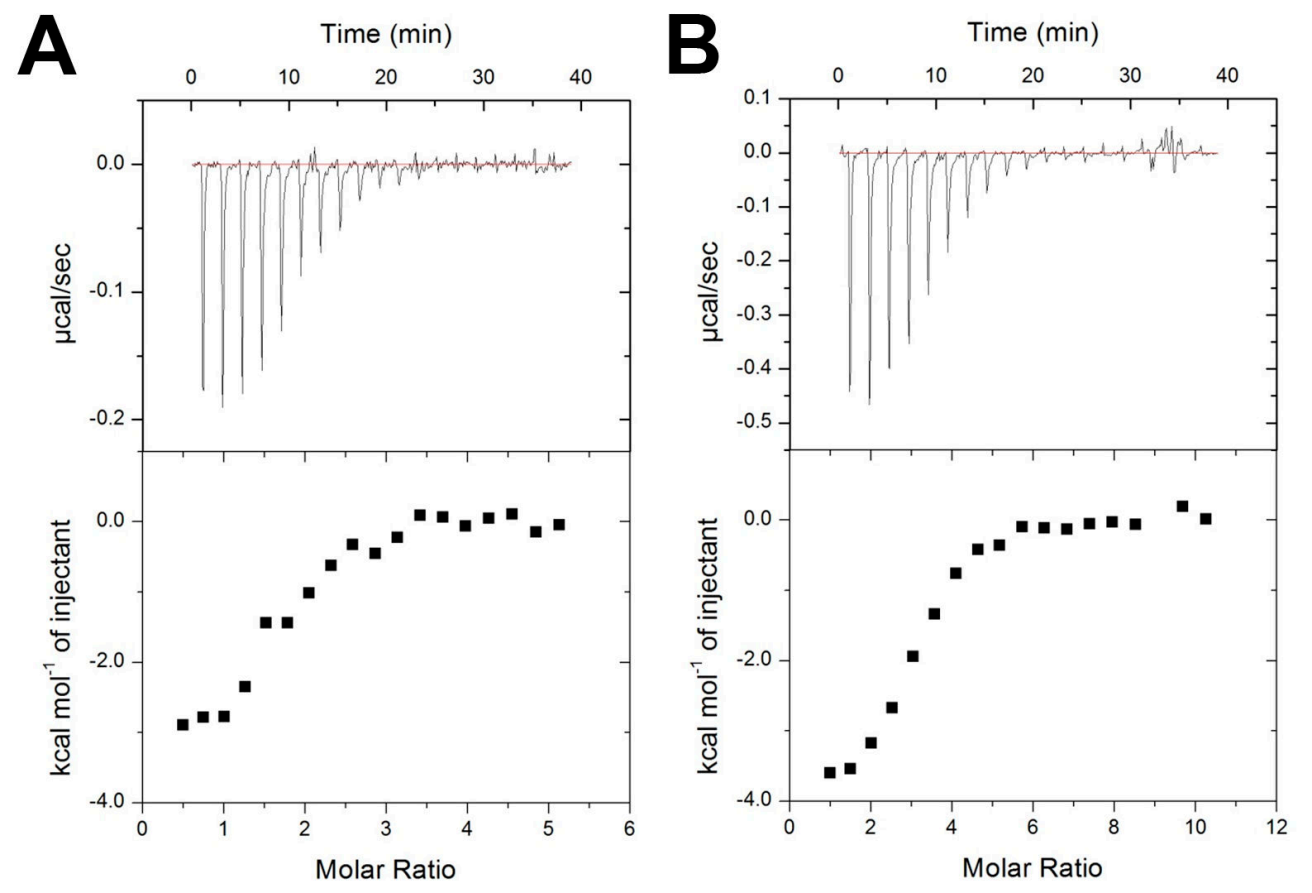
Isothermal titration of wild type/mutant ECP with Hep6. Raw ITC data of binding affinities between Hep6 and (A) wtECP and (B) double mutant ECP Q40A/H64A. Top panel: nineteen injections of Hep6 solution were titrated into protein in ITC cell. The area of each injection peak corresponded to the total heat released form that injection. Bottom panel: binding isotherm for heparin interaction with wtECP/double mutant ECP (Q40A/H64A), the integrated heat was plotted against the stoichiometry of 10.

Binding affinities of wtECP and mutant ECPs with high molecular weight heparin (HMWH), LMWH and Hep6 were measured with isothermal titration calorimetry (ITC) at 25 °C. Integrated heat effect was analyzed by means of non-linear regression using MicroCal Origin software. Dissociation constant (*K*_D_), stoichiometry value (N), enthalpy (Δ*H*) and entropy (as *T*ΔS) changes were obtained. Negative power deflections were observed throughout titration, indicating that the binding was exothermic (Figure 5). The *K*_D_ value between wtECP and HMWH was 16.4 nM, which corresponded to a free energy (ΔG) of -10.3 kcal/mol. Regarding the mutant ECPs, *K*_D_ of HMWH to ECP Q40A and H64A was 22.6 nM (ΔG=-10.2 kcal/mol) and 30.9 nM (ΔG=-10.4 kcal/mol), respectively. In the case of ECP Q40A/H64A double mutant, its *K*_d_ value to HMWH doubled (34.7 nM, ΔG=-10.2 kcal/mol), whereas ECP R105A showed very similar binding (17.9 nM, ΔG=-10.5 kcal/mol) to wtECP (Table 2). These results indicated that Gln^40^ and His^64^, but not Arg^105^, contributed to ECP-HMWH interaction. 

**Table 2:** Thermodynamic parameters for interaction between wild type/mutant ECP and heparin derivatives.

Heparin	Protein	*K*_D_ (nM)	Δ*H* (kcal/mol)	*T*ΔS (kcal/mol)	N (sites)	ΔG (kcal/mol)
	wtECP	2025.1 ± 506.3	-3 ± 0.2	4.8 ± 0.1	1.77 ± 0.1	-7.8 ± 0.1
Hep6	Q40A	2597.4 ± 870.7	-4.4 ± 0.3	3.2 ± 0.2	1.7 ± 0.1	-7.6 ± 0.2
(1,800 Da)	H64A	2164.5 ± 1072.9	-5.8 ± 0.4	2 ± 0.3	2.74 ± 0.3	-7.7 ± 0.4
	Q40A/H64A	2984 ± 536	-3.9 ± 0.2	3.7 ± 0.1	2.45 ± 0.1	-7.5 ± 0.1
	R105A	2057.6 ± 541.9	-4.4 ± 0.2	3.3 ± 0.1	2.81 ± 0.1	-7.9 ± 0.2
	wtECP	51.3 ± 7.7	-37.2 ± 0.5	-27.1 ± 0.9	0.33	-10.2 ± 0.2
LMWH	Q40A	124.4 ± 13.2	-38.4 ± 1.6	-29.2 ± 1.5	0.36	-9.2 ± 0.3
(~5,000 Da)	H64A	78.7 ± 23.6	-41.8 ± 1.5	-32.2 ± 1.3	0.41	-9.5 ± 0.5
	Q40A/H64A	147.9 ± 16.9	-35.5 ± 0.4	-26.2 ± 1.3	0.52	-9.3 ± 0.1
	R105A	33.3 ± 5.2	-36.6 ± 0.3	-32.5 ± 2.2	0.52	-9.9 ± 0.3
	wtECP	16.4 ± 3.1	-92.9 ± 1.9	-82.6 ± 5.6	0.06	-10.3 ± 0.2
HMWH	Q40A	22.6 ± 4.8	-103.6 ± 1.8	-93 ± 7.5	0.14	-10.2 ± 0.1
(~10,000 Da)	H64A	30.9 ± 8	-116.8 ± 2.3	-106.4 ± 12.8	0.19	-10.4 ± 0.4
	Q40A/H64A	34.7 ± 13.1	-90 ± 2.7	-79.9 ± 13.6	0.22	-10.2 ± 0.3
	R105A	17.9 ± 6.4	-113.6 ± 2.9	-103.1 ± 10.4	0.32	-10.5 ± 0.1

All experiments were performed at 25 °C in PBS, pH 7.4. Data are reported as the mean of three separate titrations ± SD. Hep6, heparin hexasaccharide.

Since HMWH is heterogeneous mixture, and has varying molecular weights from 5,000 Da to over 40,000 Da, binding affinity of ECPs to LMWH (1,800-7,500 Da) and Hep6 (1,800 Da) was further tested. As expected from the above-mentioned studies, the ECP-LMWH interaction was weaker than ECP-HMWH interaction in all cases (Table 2). *K*_D_ value of LMWH to wtECP was 51.3 nM (ΔG=-10.2 kcal/mol), and that to single mutant ECP Q40A and H64A were respectively 124.4 nM (ΔG=-9.2 kcal/mol) and 78.7 nM (ΔG=-9.5 kcal/mol). Besides, double mutant ECP Q40A/H64A showed almost three times weaker binding (147.9 nM, ΔG=-9.3 kcal/mol) than wtECP (51.2 nM, ΔG=-10.2 kcal/mol). Interestingly, although mutant ECP R105A showed similar HMWH binding affinity as wtECP, the LMWH binding affinity (33.3 nM, ΔG=-9.9 kcal/mol) was slightly stronger than wtECP.

For shorter heparin ligands, wtECP-Hep6 interaction showed much weaker *K*_D_ value (2 μM, ΔG=-7.8 kcal/mol) than that of LMWH and HMWH (Figure 5A, Table 2). Moreover, *K*_D_ values of Hep6 to mutant ECP Q40A, H64A, R105A and Q40A/H64A were 2.6 μM (ΔG=-7.6 kcal/mol), 2.2 μM (ΔG=-7.7 kcal/mol), 2.1 μM (ΔG=-7.9 kcal/mol) and 3 μM (ΔG=-7.5 kcal/mol), respectively (Table 2). Consistent with long-chain heparin, double mutant ECP Q40A/H64A showed weaker binding to Hep6 than wtECP (Figure 5B), but binding affinity of ECP R105A to Hep6 was similar to that of wtECP. The results indicated that Gln^40^ and His^64^ participated in the interaction between wtECP and different length of heparin molecule, and Arg^105^ might not be important for heparin oligosaccharide.

Among all types of molecular interactions, vDW force, hydrogen bonding, and ionic interaction mainly contribute to the enthalpy term. Besides, changes in conformational and hydrophobic interactions certainly contribute to the entropy term [45]. A high enthalpy term was observed in interaction between wtECP and HMWH (ΔH=-92.9 kcal/mol and TΔS=-82.6 kcal/mol) as well as interaction between wtECP and LMWH (ΔH=-37.2 kcal/mol and TΔS=-27.1 kcal/mol), clearly indicating that strong ionic interactions between the side chains of basic residues on ECP and the sulfo and carboxyl groups on heparin played major roles. Unlike wtECP interaction model with long-chain heparin, HMWH and LMWH, wtECP-Hep6 interaction was less enthalpical (ΔH=-3.28 kcal/mol and TΔS=4.47 kcal/mol), implying that long-chain heparin (HMWH and LMWH) made more contacts with ECP than Hep6 did.

### Requirement of sulfo groups on heparin for binding to ECP

**Figure 6:**
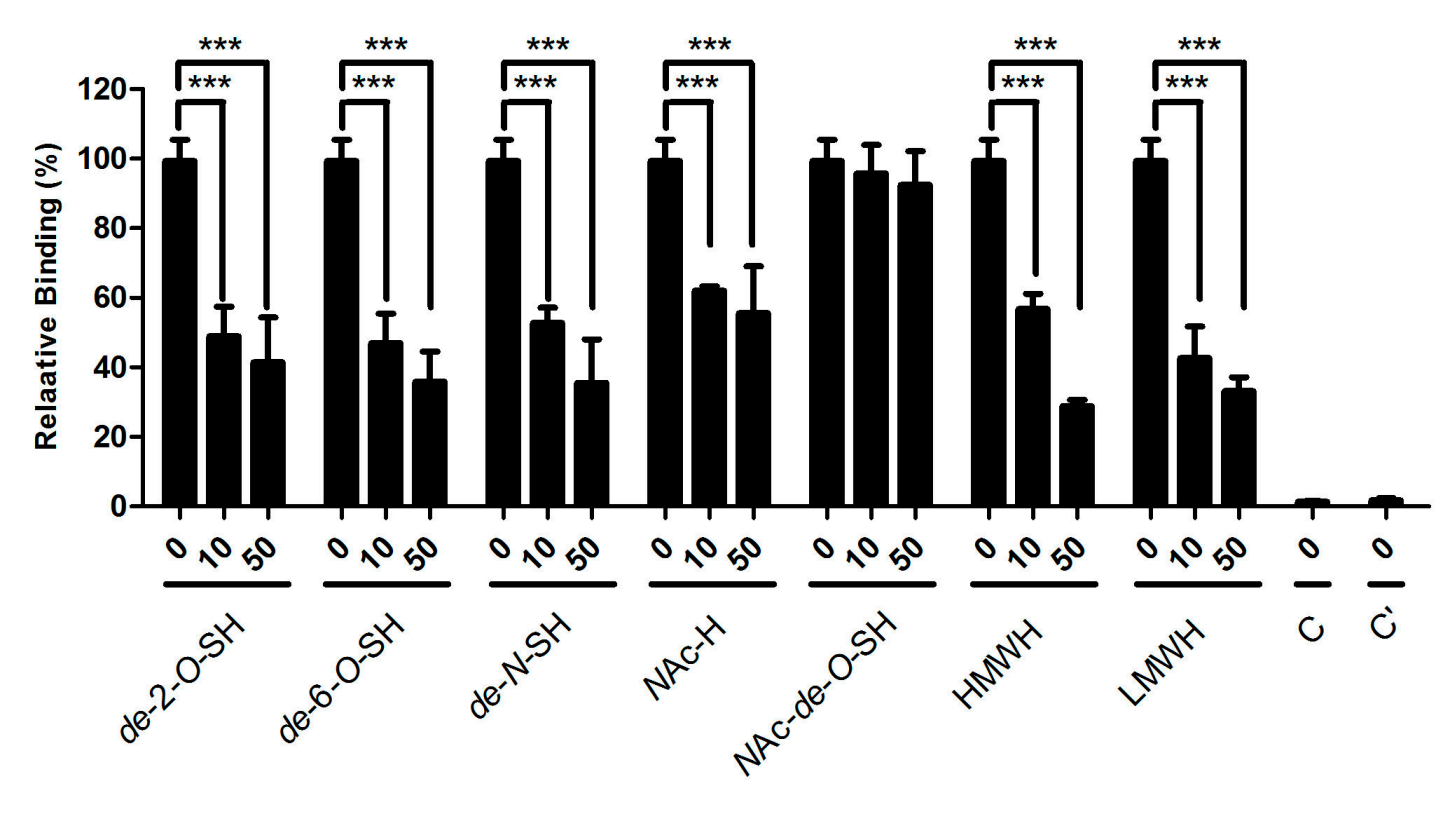
Involvement of sulfo groups in heparin binding to ECP. Beas-2B cells were pre-incubated with different heparin derivatives in RPMI 1640 medium at 4 °C for 30 min, and treated with 0.4 μM wtECP at 4 °C for an additional 1 h. The levels of bound proteins were determined by cell ELISA, and the amount of wtECP bound to Beas-2B cells without pretreatment with heparin oligosaccharide was set to 100%. C, untreated. C’, treated EG2 antibody only. The data represented three independent experiments and the error bar was shown as SD. ***, *P* < 0.001.

The contribution of *O*-sulfo and *N*-sulfo groups of heparin in wtECP-heparin interaction was estimated by comparing calculated binding energy of *de*-*O*-sulfated and *N*-acetylated Hep6 to that of the parental oligosaccharide, [GlcNS(6S)-IdoA(2S)]_3_ (Table S6). Our data suggested that these sulfo groups accounted for around 40% of total binding energy of the parental heparin oligosaccharide, and that *N*-sulfo group might have a larger contribution than *O*-sulfo group. These predictions were further confirmed by competitive cell ELISA (Figure 6, Table 1). Beas-2B cell binding activity of wtECP was inhibited by addition of heparin derivatives including *de*-2-*O*-sulfated heparin (*de*-2-O-SH), *de*-6-*O*-sulfated heparin (*de*-6-O-SH), *N*-acetyl heparin (*N*Ac-H), *N-*acetyl-*de*-*O*-sulfated heparin (*N*Ac-*de-O*-SH), HMWH and LMWH. Among these heparin derivatives, only *N*Ac-*de-O*-SH at a concentration of 50 μg/ml showed negligible levels of inhibitory effect (8%), suggesting that sulfo groups were important for wtECP-heparin interaction. Other heparin derivatives including *de*-2-O-SH, *de*-6-O-SH, NAc-H, HMWH, and LMWH showed significant inhibition of cellular binding activity to ECP, respectively 59%, 64%, 44%, 71%, and 67%, suggesting that both *N*- and *O*-sulfo groups on heparin contributed to the interaction with ECP. 

### Binding of ECP to Hep12

**Figure 7:**
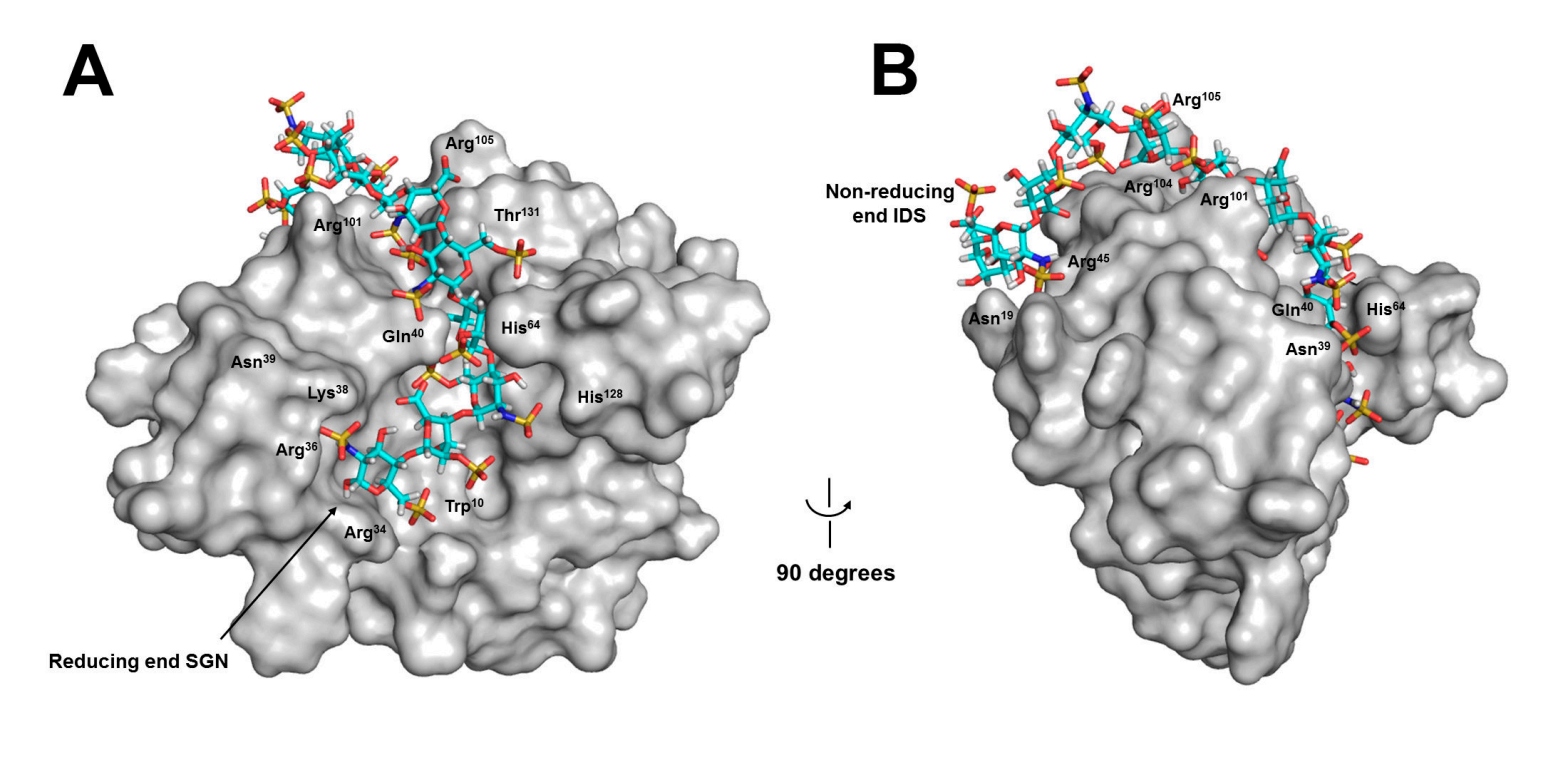
Predicted wtECP-Hep12 interaction. Hep12 bound to wtECP with the lowest energy. The ligand was shown in stick. (A) and (B) respectively represented a front and a side view of wtECP-Hep12 complex. Amino acids that interacted with the bound Hep12 on the surface were indicated by residue numbers. SGN, 6-*O*-sulfated, *N*-sulfated glucosamine; IDS, 2-*O*-sulfated iduronic acid.

Potential binding site for a longer ligand, Hep12 [GlcNS(6S)-IdoA(2S)]_6_, was investigated by a global search. The first 6 sugars from the reducing end bound to exactly identical location as Hep6 did (Figure 7A). Residues from sugars 7 to 12 were likely to interact with ECP at an extended part of the cavity on ECP surface by making additional interactions with Asn^19^, Arg^45^, Arg^101^ and Arg^104^ (Figure 7B). Among these residues Arg^101^ and Arg^104^ were located at HBR3*ecp* [22], and Asn^19^ and Arg^45^ were located at a position close to non-reducing end of Hep12, suggesting that HBR3*ecp* might participate in the interaction between wtECP and long-chain heparin. 

## Discussion

ECP was derived from an ECP/EDN precursor gene about 30 million years ago at a high evolutionary rate among primate genes [46]. To investigate conservation of HBR sequences as well as Gln^40^ and His^64^ residues in all primate ECP/EDNs, multiple sequences originating from different primates including *Homo sapiens*, *Gorilla Gorilla*, *Pan troglodytes*, *Pongo pygmaeus*, *Macaca fascicularis*, and *Macaca nemestrina* were aligned. Interestingly, Figure S2 showed that ECP of all six species had conserved Gln^40^ residues, but only higher primates *P*. *troglodytes* and *G*. *Gorilla*, the closest living relatives of humans [47], had His^64^ residues at comparative location. *P. pygmaeus* and *M. fascicularis* ECPs had Arg^64^, and *M. nemestrina* ECP had Ser^64^ as human EDN. Regarding the corresponding residues of EDN, all primate EDN sequences possessed highly conserved Gln^40^ and Ser^64^ pairs. Here the unique Gln^40^-His^64^ clamp might be evolved to stabilize heparin molecules through ring-ring interaction. Poor ribonucleolytic activity of ECP may be in part due to close vicinity and longer side chain of His^64^ to the catalytic site of RNase. Similar result was observed in HBR1*ecp* motif that only higher primates *P*. *troglodytes* and *G*. *Gorilla* ECPs had characteristic ^34^RWRCK^38^ sequence, whereas other species had identical HBR1 sequences as HBR1*edn* ^34^,QRRCK^38^ (Figure S2). Since HBR1*edn* fit conventional XBBXBX motif, it might play a major role in heparin binding by RNase 2 family. Within HBR1*ecp* motif, Trp^35^ was demonstrated to be important for ECP interaction with membrane [32]. Higher primate ECPs apparently had point mutations in HBR1 sequence from EDN pattern Gln^34^ and Arg^35^ to Arg^34^ and Trp^35^ to increase lipid binding and cell penetrating activities [48], which in turn resulted in unique tertiary structure forming Gln^40^ and His^64^ clamp to strengthen heparin binding activity of ECP (Figure 4B). Regarding HBR3*ecp* involved in Hep12 binding, except *P. pygmaeus* all five primate ECPs had the same sequence ^101^,RPGRR^105^. Interestingly, EDN in higher primates including *H. sapiens*, *P*. *troglodytes* and *G*. *Gorilla* had exactly identical basic residue composition in HBR3*edn*, suggesting that this region might also involve in long-chain heparin binding by EDN. In comparison with HBR1 and HBR3, HBR2 showed lower sequence identity in primate ECPs, and only *H. sapiens*, *G*. *Gorilla* and *M. fascicularis* ECPs had three basic residues in this region. However, identical sequences were conserved in HBR2*edn* of higher primates (Figure S2). Since mutant recombinant human ECP with Ala replacement in HBR2*ecp* was reported to decrease 20% of heparin binding activity [22], this region might play an auxiliary role in heparin binding. These results strongly suggested that key heparin binding residues in human ECP were gained along with species evolution of primate eosinophil RNases.

Gln^40^ and His^64^ have been previously predicted to involve in Hep6 binding by making hydrogen bonds in a rigid ECP structure (PDB: 1DYT) [42]. However, these two residues were fixed in a wide distance of 8.1 Å at top edge of the basic cavity to interact with heparin molecule. In our model, basic residues including Arg, Lys and His as well as polar residues Asn and Gln were set to be flexible, which allowed Gln^40^ and His^64^ to rotate and form a heparin binding clamp at a closer distance of 6.5 Å. Human ECP and trisaccharide heparin mimetic complex structure (PDB: 2LVZ) also indicated that Gln^40^ and His^64^ were involved in ligand binding [32]. Since the ligand was as short as only three saccharide units, it might have high priority to bind with the residues located at the central basic cavity on ECP surface, including HBR1*ecp*. However, the distance between these Gln^40^ and His^64^ was much wider (>15 Å) than that of our model. Regarding the complex structures of ECP to sulfate anions (PDB: 4A2O) [29] and 2’,5’-ADP (PDB: 1H1H) [30], the distance between Gln^40^ and His^64^ was respectively 7.1 Å and 11.8 Å, too far to interact with the ligands.

HBR1*ecp*, ^34^RWRCK^38^, was demonstrated to participate in ECP-heparin interaction [21], and a 10-amino acid peptide containing this region, ^32^NYRWRCKNQN^41^, (CPP*ecp*), showed not only cell surface heparan sulfate proteoglycans binding but also cell penetrating activities [48]. In contrast, the corresponding region in EDN, ^32^NYQRRCKNQN^41^, fit conventional heparin binding motif XBBXBX yet demonstrated only very weak heparin binding and cell penetrating activities [48]. Superimposition of Hep6 bound pose of ECP to that of EDN by fitting Cα atoms of residues 32 to 41 revealed that all the amino acids except Lys^38^ in this region oriented in similar angles (Figure S3). In HBR1*ecp* Arg^34^ and Arg^36^ bound Hep6 by ionic interactions, and Cys^37^ and Lys^38^ were also observed to interact with Hep6 by hydrogen bond and vDW force. In the case of HBR1*edn*, our previous docking simulation data indicated that Hep6 was stabilized by making ionic bonds with Arg^36^ and Lys^38^ and a vDW interaction with Gln^34^ (Table S4) [43], suggesting that HBR1 in both ECP and EDN played major roles in heparin binding. Interestingly, Arg^35^ of EDN was not involved in any interaction with Hep6, different from the corresponding Trp^35^ of ECP which involved in cell binding and penetrating activities [32,48]. In should be noted that HBR1*edn* was located at a loop, unlike most XBBXBX motif which tended to form β-strand conformation facilitating interaction with negatively charged sulfo groups on heparin [40]. It was thus possible that the side chain of three basic residues in HBR1*edn* might not orient in the same face to interact with heparin molecule even if HBR*edn* fit the conventional heparin binding motif in primary sequence. In addition to HBR1, Gln^40^ of both human ECP and EDN contacted Hep6 through hydrogen bonds and vDW forces, and other residues including Asn^39^ in both ECP and EDN and Asn^41^ in ECP interacted with heparin molecule by vDW forces (Table S1, Table S3). Interestingly, in the absence of Gln^40^, the cell penetrating activity of CPP*ecp* significantly reduced [48], strongly suggesting that Gln^40^ interaction with heparin was critical for unique features of ECP and CPP*ecp*. Therefore, evolutionarily gained sequence variation in residues 32 to 41 of ECP from EDN precursor apparently correlated with distinct structural features and biological functions.

Our ITC data revealed that binding affinities of all heparin variants to ECP Q40A/H64A was 1.5 to 3 times weaker than that of wtECP, suggesting that Gln^40^ and His^64^ clamp contributed to ECP-heparin interaction significantly (Table 2). However, binding affinity of wtECP to Hep6 was much weaker than those of long-chain heparin molecules (LMWH and HMWH), unlike other heparin binding proteins, extracellular superoxide dismutase and fibroblast growth factor-1 (FGF-1) which had only one ligand binding site showing similar *K*_*D*_ values to different lengths of heparin molecules [49,50]. Besides, our competitive cell ELISA data also indicated that *N*Ac-*de*-*O*-SH completely devoid of *O*-sulfo group with markedly diminished *N*-sulfo groups could not inhibit interaction between wtECP and Beas-2B cells (Figure 6, Table 1), suggesting that ionic interactions played crucial roles for ECP interaction with heparin oligosaccharides. FGF family members were reported to interact with heparin molecule through specific position of sulfo groups, including 6-*O* sulfated heparin (FGF-1) [51], 2-*O* sulfated heparin (FGF-2) [52] and 3-*O*-sulfo group which rarely appears in heparin/HS (FGF-7) [53]. In the case of ECP, no significant inhibitory effect was observed in heparin derivatives with selectively modified sulfo groups, NAcH, *deN*-SH, *de*2*O*-SH, and *de*6*O*-SH, suggesting that ECP might not interact with heparin through specific position of sulfo groups. 

 The interactions between ECP and HMWH/LMWH were enthalpy driven, however, positive values of *T*ΔS were observed only in interactions of wtECP and mutant ECPs with Hep6 (Table 2). Such phenomenon was also reported in the interaction between short-chain heparin and extracellular superoxide dismutase [49]. Since Hep6 had less glycosidic bonds than HMWH and LMWH, its structure was more rigid than long-chain heparins, leading to loss in degree of freedom in Hep6 upon binding with wtECP in a less extent than HMWH and LMWH. Moreover, contribution of released sodium and water ions might account for less negative entropy contribution of ECP interaction with Hep6.

Since the size of heparin, even LMWH, is very large with many rotatable bonds, accurate docking is difficult. Here a stepwise method was used to obtain computer model for ECP-Hep12 interaction. The first step was identical to ECP-Hep6 to set all Arg, Lys, His, Asn and Gln to be flexible, and allowed all rotatable bonds to rotate during computation. Hep6 was docked to ECP allowing the flexible residues to find optimal conformations. In the second step, Hep6 (6 disaccharide units) was docked to ECP that had those residues with the side chains optimized in the first step. This two-step method allowed us to obtain docked poses between ECP and Hep12. Our model showed that both HBR1*ecp* and HBR3*ecp* interacted with Hep12, suggesting a possibility that a larger heparin chain, such as HMWH/LMWH might simultaneously interact with more than one HBR of ECP.

In conclusion, we have demonstrated that high affinity of ECP to heparin/HS may not only be contributed by conventional positively charged residues but also by a unique Gln^40^ and His^64^ clamp. Realizing of the roles of key functional residues of ECP further provides informative clues elaborating versatile activities of this human RNaseA family member.

## Supporting Information

Figure S1 **Purification of wtECP and mutant ECPs.** In each lane 3 μg protein was loaded on a 15% (w/v) SDS-PAGE. The molecular weight of marker was labeled as M and indicated at left. M: marker; lane 1: wtECP, lanes 2-5: mutant ECP Q40A, H64A, R105A, Q40A/H64A.
(TIF)

Figure S2 **Sequence alignment of primate human eosinophil RNases and HBRs.** Amino acid sequences were aligned using *Clustal* *X2* [54]. Putative heparin binding regions (HBRs) were framed. Fully conserved amino acids were indicated by asterisk (*), highly similar amino acids were indicated by colon (:), and weakly similar amino acids were indicated by dot (.).
(TIF)

Figure S3 **Structure comparison between residues 32 to 41 in human ECP and EDN.** Superimpose analysis was performed using Lsqkab. Hep6 bound pose of ECP (orange) was superimposed on that of EDN (pink) by fitting Cα atoms of residues 32 to 41. Side chains of amino acids in this region were shown in stick with indicated numbers. The Hep6 that interacting with ECP and EDN was respectively shown in red and blue lines.
(TIF)

Table S1 **Interaction between wild type ECP and Hep6.**
(DOCX)

Table S2 **Calculated binding energy of Hep6 to various ECP mutants and contribution of individual amino acid.**
(DOCX)

Table S3 **Interaction between wild type EDN and Hep6 [43].**
(DOCX)

Table S4 **Calculated binding energy of Hep6 to various EDN mutants and contribution of individual amino acid.**
(DOCX)

Table S5 **RNase activity of wild type/mutant ECP.**
(DOCX)

Table S6 **Calculated binding energy of wild type ECP to various heparin derivatives and contribution of *O*-sulfate and *N*-sulfate groups on Hep6.**
(DOCX)
