# Supplementary figures and images for: Functional Characterization of ECP-Heparin Interaction: A Novel Molecular Model
Source: PLoS One. 2013 Dec 11;8(12):e82585. doi: 10.1371/journal.pone.0082585 (PMC3859622; doi:10.1371/journal.pone.0082585)

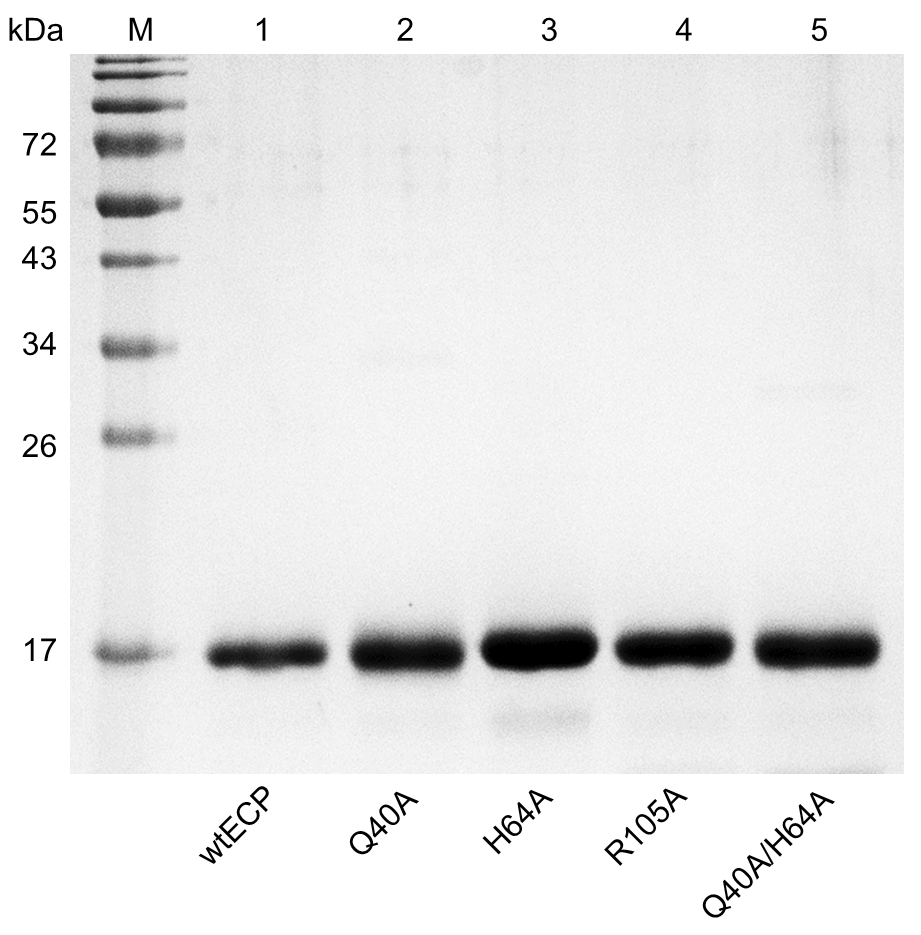

Supplement: Figure S1 — Purification of wtECP and mutant ECPs. In each lane 3 μg protein was loaded on a 15% (w/v) SDS-PAGE. The molecular weight of marker was labeled as M and indicated at left. M: marker; lane 1: wtECP, lanes 2-5: mutant ECP Q40A, H64A, R105A, Q40A/H64A. (TIF) [file pone.0082585.s001.tif]

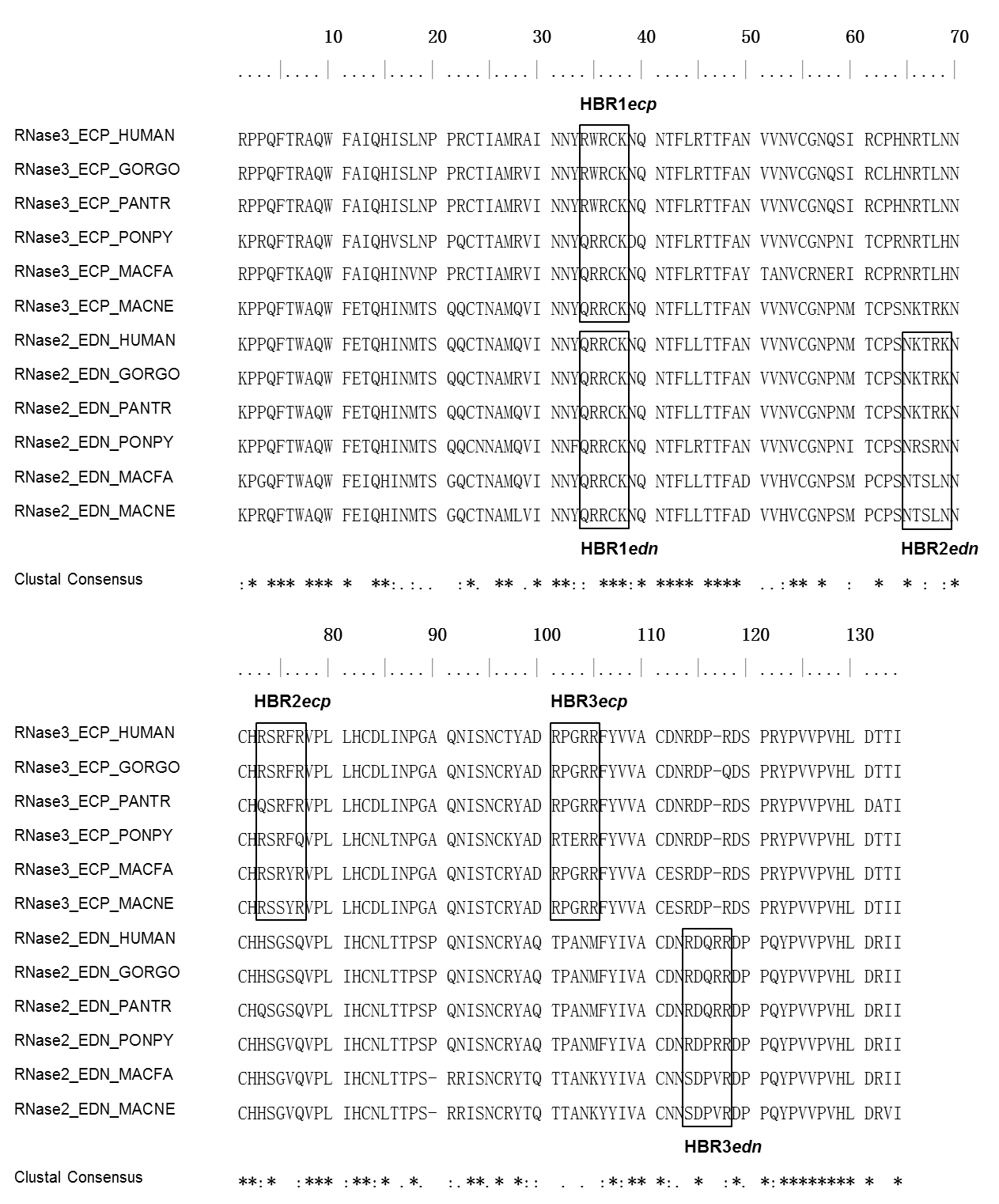

Supplement: Figure S2 — Sequence alignment of primate human eosinophil RNases and HBRs. Amino acid sequences were aligned using Clustal X2 [54]. Putative heparin binding regions (HBRs) were framed. Fully conserved amino acids were indicated by asterisk (*), highly similar amino acids were indicated by colon (:), and weakly similar amino acids were indicated by dot (.). (TIF) [file pone.0082585.s002.tif]

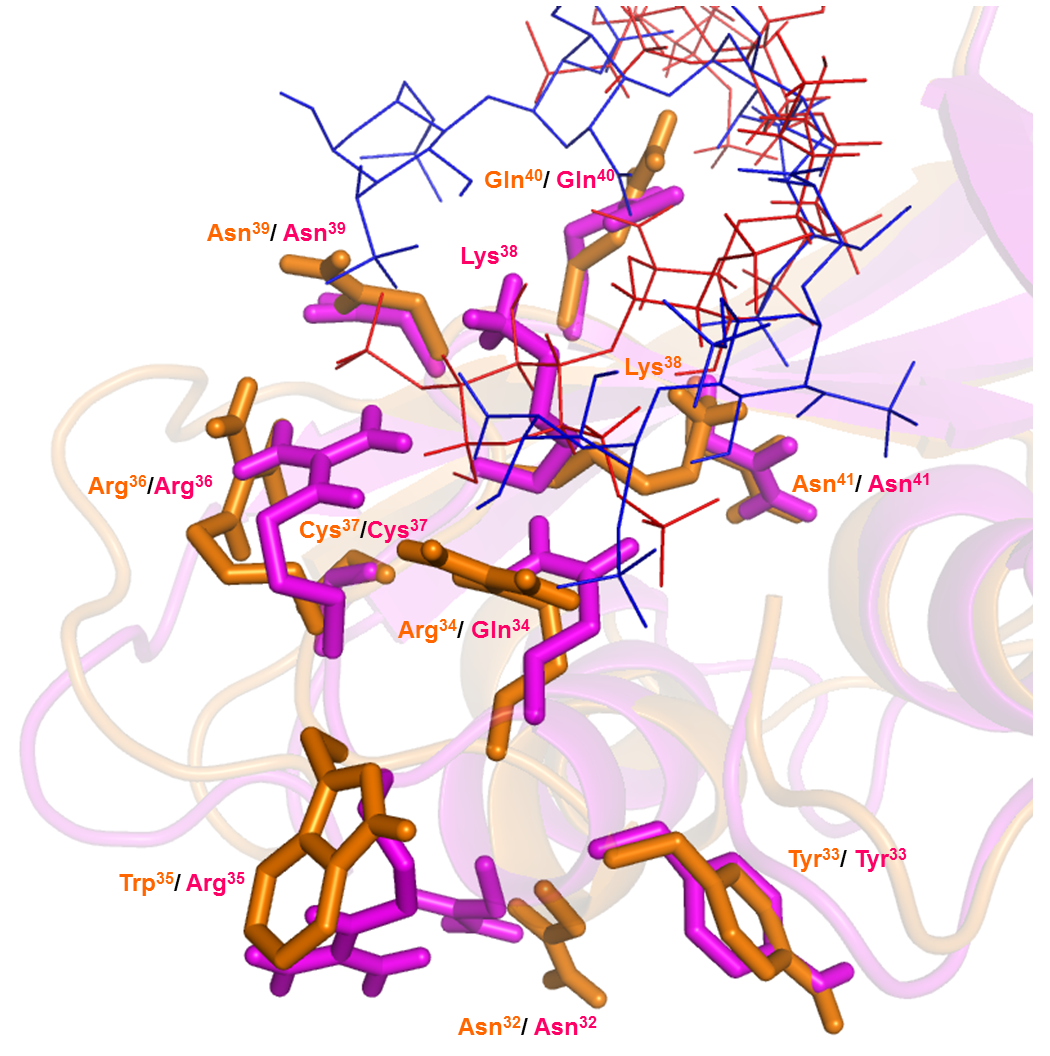

Supplement: Figure S3 — Structure comparison between residues 32 to 41 in human ECP and EDN. Superimpose analysis was performed using Lsqkab. Hep6 bound pose of ECP (orange) was superimposed on that of EDN (pink) by fitting Cα atoms of residues 32 to 41. Side chains of amino acids in this region were shown in stick with indicated numbers. The Hep6 that interacting with ECP and EDN was respectively shown in red and blue lines. (TIF) [file pone.0082585.s003.tif]
