# Supplementary material for: Functional Characterization of ECP-Heparin Interaction: A Novel Molecular Model
Source: PLoS One. 2013 Dec 11;8(12):e82585. doi: 10.1371/journal.pone.0082585 (PMC3859622; doi:10.1371/journal.pone.0082585)
Supplement: Table S1 — Interaction between wild type ECP and Hep6. (DOCX) [file pone.0082585.s004.docx]

**Table S1. Interaction between wild type ECP and heparin hexasaccharide**

| Residue | Atom/group | Type of interaction | Residue | Atom/group |
| --- | --- | --- | --- | --- |
| Trp^10^ | N1 | H-bond | SGN1 | 6-*O*-sulfate |
| His^15^ | τ-nitrogen | vDW | SGN3 | C6 and oxygen of 6-*O*-sulfate |
| Arg^34^ | Guanidino group | Ionic | SGN1 | 6-*O*-sulfate |
|  | δ-nitrogen | vDW | SGN1 | Oxygen of C1-OH |
| Arg^36^ | Guanidino group | Ionic | SGN1 | *N*-sulfate |
| Cys^37^ | Backbone carbonyl oxygen | H-bond | SGN1 | C1-OH |
|  | Backbone carbonyl carbon | vDW | SGN1 | C1-OH |
| Lys^38^ | ε-NH_2_ | vDW | IDS2 | C3-OH |
| Asn^39^ | Backbone amide nitrogen | vDW | SGN1 | Hydrogen of NH within N-sulfate |
|  | Backbone amide hydrogen | vDW | SGN1 | Nitrogen of NH within *N*-sulfate |
|  | NH_2_ of side-chain amide group | vDW | SGN1 | Oxygen of *N*-sulfate |
| Gln^40^ | δ-carbon | vDW | SGN5 | Nitrogen of *N*-sulfate |
|  | Oxygen of side-chain amide group | vDW | SGN5 | Nitrogen of *N*-sulfate |
|  | Nitrogen of side-chain amide group | vDW | SGN5 | Nitrogen of *N*-sulfate |
|  | Nitrogen of side-chain amide group | vDW | IDS4 | Oxygen and sulfur of 2-*O*-sulfate |
|  | Hydrogen of side-chain amide group | H-bond | IDS2 | C5 carboxyl group |
| Asn^41^ | α-carbon | vDW | SGN3 | Sulfur of 6-*O*-sulfate |
| Thr^42^ | Backbone amide nitrogen | vDW | SGN3 | Sulfur of 6-*O*-sulfate |
|  | Hydrogen of backbone amide group | H-bond | SGN3 | Oxygen of 6-*O*-sulfate |
| His^64^ | C4 | vDW | IDS4 | Hydrogen of C3-OH |
|  | C5 | vDW | SGN3 | C3-OH |
|  | π-nitrogen | vDW | IDS4 | Hydrogen of C3-OH |
|  | C2 | vDW | SGN5 | C2 and C3 |
|  | Hydrogen on tele-nitrogen | vDW | IDS4 | 2-*O*-sulfate |
| Arg^105^ | Carbon of guanidino group | vDW | IDS6 | Oxygen of C4-OH |
|  | ω-Nitrogen of guanidino group | vDW | IDS6 | Oxygen of C5-carboxyl group |
|  | Hydrogen of guanidino group | H-bond | IDS6 | Oxygen of C5-carboxyl group |
|  | ω’-Nitrogen of guanidino group | vDW | IDS6 | Oxygen of 2-*O*-sulfate |
|  | Hydrogen of guanidino group | vDW | IDS6 | C5, oxygen of C5-carboxyl group,  and ring oxygen |
| Leu^129^ | Backbone carbonyl oxygen | vDW | SGN3 | C4 |
|  | β-carbon | vDW | SGN3 | C6 |
| Thr^131^ | Hydrogen of side-chain OH group | H-bond | SGN5 | Oxygen of 6-*O*-sulfate |

H-bond, hydrogen bond; vDW, van der Waals force; SGN, 6-*O*-sulfated, *N*-sulfated glucosamine; IDS, 2-*O*-sulfated iduronic acid.
