# Supplementary material for: Functional Characterization of ECP-Heparin Interaction: A Novel Molecular Model
Source: PLoS One. 2013 Dec 11;8(12):e82585. doi: 10.1371/journal.pone.0082585 (PMC3859622; doi:10.1371/journal.pone.0082585)
Supplement: Table S2 — Calculated binding energy of Hep6 to various ECP mutants and contribution of individual amino acid. (DOCX) [file pone.0082585.s005.docx]

**Table S2. Calculated binding energy of heparin hexasaccharide to various ECP mutants and contribution of individual amino acid**

|  | Binding energy (kcal/mol) | A.A. | Contribution (kcal/mol) |
| --- | --- | --- | --- |
| Wild-type | -9.24 |  |  |
| W10A | -9.00 | W10 | -0.24 |
| H15A | -9.13 | H15 | -0.11 |
| R34A | -8.68 | R34 | -0.56 |
| R36A | -8.94 | R36 | -0.30 |
| C37A | -9.23 | C37 | -0.01 |
| K38A | -8.86 | K38 | -0.38 |
| N39A | -9.05 | N39 | -0.19 |
| Q40A | -8.32 | Q40 | -0.92 |
| N41A | -9.17 | N41 | -0.07 |
| T42A | -9.06 | T42 | -0.18 |
| H64A | -8.69 | H64 | -0.55 |
| R105A | -8.56 | R105 | -0.68 |
| L129A | -9.10 | L129 | -0.14 |
| T131A | -8.96 | T131 | -0.28 |
