# Supplementary material for: Functional Characterization of ECP-Heparin Interaction: A Novel Molecular Model
Source: PLoS One. 2013 Dec 11;8(12):e82585. doi: 10.1371/journal.pone.0082585 (PMC3859622; doi:10.1371/journal.pone.0082585)
Supplement: Table S3 — Interaction between wild type EDN and Hep6 [43]. (DOCX) [file pone.0082585.s006.docx]

**Table S3.** **Interaction between wild type EDN and heparin hexasaccharide [**[**43**](#_ENREF_43)**]**

| Residue | Atom/group | Type of interaction | Residue | Atom/group |
| --- | --- | --- | --- | --- |
| Lys^1^ | α-NH_3_^+^ | H-bond/Ionic | IDS6 | Oxygen of 2-*O*-sulfate |
| Trp^7^ | C-4 | vDW | SGN5 | Oxygen of *N*-sulfate |
|  | C-5 | vDW | SGN5 | C-1 |
| Trp^10^ | N-1 | H-bond | SGN5 | Oxygen of 3-OH |
| Gln^14^ | Amide NH_2_ | vDW | IDS4 | Oxygen of 2-*O*-sulfate |
|  | Amide NH_2_ | vDW | SGN5 | Nitrogen, hydrogen, and oxygen of *N*-sulfonyl group |
| His^15^ | Hydrogen of τ-nitrogen | H-bond | SGN5 | Oxygen of *N*-sulfonyl group |
|  | C-2 | vDW | IDS4 | Oxygen of 2-*O*-sulfate |
| Gln^34^ | Carbon of side-chain amide group | vDW | IDS6 | Ring oxygen |
| Arg^36^ | δ- and ω-Nitrogen | H-bond/Ionic | IDS6 | Oxygen of C-6 carboxyl group |
|  | ω-Nitrogen | Ionic | SGN1 | Oxygen of *N*-Sulfonyl group |
|  | C-5 | vDW | SGN1 | Oxygen of *N*-Sulfonyl group |
| Lys^38^ | ε-NH_3_^+^ group | Ionic | SGN1 | Oxygen of *N*-Sulfonyl group |
|  | Hydrogen of ε-NH_3_^+^ group | vDW | SGN1 | Oxygen of C3-OH group |
| Asn^39^ | Hydrogen of side-chain amide group | vDW | SGN1 | Oxygen of sulfonyl group |
| Gln^40^ | C-3 | vDW | IDS2 | C-1 |
|  | C-4 | vDW | IDS2 | C-1 and oxygen of 2-*O*-sulfonyl group |
|  | Nitrogen of side-chain amide group | vDW | IDS2 | C-1, C-5, and C-6 |
|  | Nitrogen of side-chain amide group | H-bond | IDS2 | Ring oxygen |
| His^129^ | C-5 | vDW | IDS4 | C-4 |
|  | C-5 | vDW | SGN5 | C-1 |
| Leu^130^ | C-1 | vDW | IDS4 | Hydrogen of C-3-OH |
|  | Backbone carbonyl oxygen | H-bond | IDS4 | C-3-OH |
| Arg^132^ | ω-Nitrogen | Ionic | SGN3 | *N*-sulfate |
|  | ω-Nitrogen | Ionic | IDS2 | 2-*O*-sulfate |

H-bond, hydrogen bond; vDW, van der Waals force; SGN, 6-*O*-sulfated, *N*-sulfated glucosamine; IDS, 2-*O*-sulfated iduronic acid.
