# Supplementary material for: Functional Characterization of ECP-Heparin Interaction: A Novel Molecular Model
Source: PLoS One. 2013 Dec 11;8(12):e82585. doi: 10.1371/journal.pone.0082585 (PMC3859622; doi:10.1371/journal.pone.0082585)
Supplement: Table S4 — Calculated binding energy of Hep6 to various EDN mutants and contribution of individual amino acid. (DOCX) [file pone.0082585.s007.docx]

**Table S4. Calculated binding energy of heparin hexasaccharide to various EDN mutants and contribution of individual amino acid**

|  | Binding energy (kcal/mol) | A.A. | Contribution (kcal/mol) |
| --- | --- | --- | --- |
| Wild-type | -9.27 |  |  |
| W7A | -8.93 | W7 | -0.34 |
| W10A | -8.88 | W10 | -0.39 |
| Q14A | -8.81 | Q14 | -0.46 |
| H15A | -8.93 | H15 | -0.34 |
| Q34A | -8.88 | Q34 | -0.39 |
| R36A | -8.71 | R36 | -0.56 |
| K38A | -8.66 | K38 | -0.61 |
| N39A | -8.98 | N39 | -0.29 |
| Q40A | -8.50 | Q40 | -0.77 |
| H129A | -9.05 | H120 | -0.22 |
| L130A | -9.19 | L130 | -0.08 |
| R132A | -8.81 | R132 | -0.46 |
