# Supplementary material for: Functional Characterization of ECP-Heparin Interaction: A Novel Molecular Model
Source: PLoS One. 2013 Dec 11;8(12):e82585. doi: 10.1371/journal.pone.0082585 (PMC3859622; doi:10.1371/journal.pone.0082585)
Supplement: Table S5 — RNase activity of wild type/mutant ECP. (DOCX) [file pone.0082585.s008.docx]

**Table S5. RNase activity of wild type/mutant ECP**

| RNase | Activity (soluble tRNA/pmol) |
| --- | --- |
| Bovine RNase A | 1.1710 ± 0.0131 |
| wtECP | 0.0049 ± 0.0001 |
| Q40A | 0.0043 ± 0.0001 |
| H64A | 0.0068 ± 0.0001 |
| Q40A/H64A | 0.0056 ± 0.0002 |
| R105A | 0.0032 ± 0.0002 |

The data represented the means of triplicate incubation with error bars shown as SD.
