# Supplementary material for: Functional Characterization of ECP-Heparin Interaction: A Novel Molecular Model
Source: PLoS One. 2013 Dec 11;8(12):e82585. doi: 10.1371/journal.pone.0082585 (PMC3859622; doi:10.1371/journal.pone.0082585)
Supplement: Table S6 — Calculated binding energy of wild type ECP to various heparin derivatives and contribution of O-sulfate and N-sulfate groups on Hep6. (DOCX) [file pone.0082585.s009.docx]

**Table S6. Calculated binding energy of wild type ECP to various heparin derivatives and contribution of *O*-sulfate and *N*-sulfate groups on heparin hexasaccharide**

|  | Binding energy (kcal/mol) | Sulfate groups | Contribution (kcal/mol) | (%) |
| --- | --- | --- | --- | --- |
| IdoA(2S)-GlcNS(6S) | -9.24 |  |  |  |
| *De*-2-*O*-sulfated Hep6 | -8.57 | 2-*O*-Sulfate | -0.67 | 7 |
| *De*-6-*O*-sulfated Hep6 | -8.00 | 6-*O*-Sulfate | -1.24 | 13 |
| *N*-Acetyl Hep6 | -7.17 | *N*-Sulfate | -2.07 | 22 |
| *N*-Acetyl-*de*-*O*-sulfated Hep6 | -5.45 | All sulfate | -3.79 | 41 |
| Other Interactions |  |  | -5.45 | 59 |

Hep6, heparin hexasaccharide.
